# Physical Cues Controlling Seasonal Immune Allocation in a Natural Piscine Model

**DOI:** 10.3389/fimmu.2018.00582

**Published:** 2018-03-22

**Authors:** Alexander Stewart, Pascal I. Hablützel, Hayley V. Watson, Martha Brown, Ida M. Friberg, Joanne Cable, Joseph A. Jackson

**Affiliations:** ^1^School of Biosciences, Cardiff University, Cardiff, United Kingdom; ^2^Faculty of Health and Medical Sciences, University of Surrey, Guildford, United Kingdom; ^3^IBERS, Aberystwyth University, Aberystwyth, United Kingdom; ^4^Flanders Marine Institute, Oostende, Belgium; ^5^Laboratory of Biodiversity and Evolutionary Genomics, Biology Department, University of Leuven, Leuven, Belgium; ^6^School of Environmental Sciences, University of Hull, Hull, United Kingdom; ^7^School of Environment and Life Sciences, University of Salford, Salford, United Kingdom

**Keywords:** *Gasterosteus aculeatus*, immunity, immunoregulation, seasonality, photoperiod, temperature

## Abstract

Seasonal patterns in immunity are frequently observed in vertebrates but are poorly understood. Here, we focused on a natural piscine model, the three-spined stickleback (*Gasterosteus aculeatus*), and asked how seasonal immune allocation is driven by physical variables (time, light, and heat). Using functionally-relevant gene expression metrics as a reporter of seasonal immune allocation, we synchronously sampled fish monthly from the wild (two habitats), and from semi-natural outdoors mesocosms (stocked from one of the wild habitats). This was repeated across two annual cycles, with continuous within-habitat monitoring of environmental temperature and implementing a manipulation of temperature in the mesocosms. We also conducted a long-term laboratory experiment, subjecting acclimated wild fish to natural and accelerated (×2) photoperiodic change at 7 and 15°C. The laboratory experiment demonstrated that immune allocation was independent of photoperiod and only a very modest effect, at most, was controlled by a tentative endogenous circannual rhythm. On the other hand, experimentally-determined thermal effects were able to quantitatively predict much of the summer–winter fluctuation observed in the field and mesocosms. Importantly, however, temperature was insufficient to fully predict, and occasionally was a poor predictor of, natural patterns. Thermal effects can thus be overridden by other (unidentified) natural environmental variation and do not take the form of an unavoidable constraint due to cold-blooded physiology. This is consistent with a context-dependent strategic control of immunity in response to temperature variation, and points to the existence of temperature-sensitive regulatory circuits that might be conserved in other vertebrates.

## Introduction

Disease risk, in humans and animals, is frequently seasonal and seasonal variation in host immune allocation (1–4) may contribute to this. Moreover, seasonal change in immune responses is often reported in vertebrates (5–9) and might constrain not just infectious disease, through effects on immuncompetence, but also autoimmune disease, through altering the tendency for immune autoreactivity. Despite this importance, the proximal controllers of seasonal variation in immunity are incompletely understood. Among the physical correlates of season, several candidates might be considered, including photoperiodic variation (7, 10, 11), the passing of time measured by an endogenous clock (9), or environmental temperature variation (7, 12, 13). However, evidence for each of these is phylogenetically patchy among vertebrates, or contradictory, and existing studies tend either to use relatively unnatural experimental regimens in the animal house, or an observational approach in the field, unable to disentangle the mass of collinear variables involved in seasonal progression.

Our aim in this study is to assess the physical cues driving seasonal immune allocation in natural populations. Importantly, we set out to bridge the gap between the animal house and the field—drawing together elements that embody the experimental control of the former, allowing strong causal inference, and the natural context of the latter. We achieved this by combining detailed monitoring of natural populations, experimental manipulations in outdoor semi-natural mesocosms and a long-term laboratory experiment using acclimatized wild animals exposed to gradual (naturalistic), rather than drastic (unnatural), seasonal photoperiodic change. In taking such an approach to photoperiodic manipulation, we reduced the possibility that very unnatural photoperiod (PP) changes might confound outcomes through the stress effects of disruption of the circadian machinery (14) or through the formation of aberrant (e.g., unnaturally prolonged) breeding phenotypes (15).

Focusing on a piscine model, the three-spined stickleback (*Gasterosteus aculeatus*), we thus ask whether major seasonal physical variables (time, light, and heat) provide the cues controlling circannual patterns in immunity in a natural environment. We chose this species as it is an intensively studied natural model (16, 17), occurring in highly seasonal mid-latitude habitats and with an annotated full genome (18) facilitating postgenomic study. In the same way that other teleosts, such as zebrafish and medaka, are increasingly used to study disease processes relevant to mammalian health (19), the three-spined stickleback – because it contains all of the central elements of adaptive immunity (20, 21)—has a general comparative relevance for immunity in other vertebrates. Even more pertinently, we have previously characterized seasonal patterns of immune gene expression in wild *G. aculeatus* populations (22), and the species has been much studied with regard to the environmental cues initiating reproduction (23–27). Stimulation of seasonal reproductive activity in *G. aculeatus* can involve a weak endogenous circannual oscillator and responses to photoperiodic and thermal cues (23–27). These control mechanisms could potentially be co-opted for the seasonal regulation of immunity.

As a reporter of phenotypic change in the immune system we measured mRNA gene expression responses that we have previously demonstrated to show seasonal variation (22, 28). Although early mRNA vs protein correlational surveys, in many organisms, led to doubts on the biological meaningfulness of mRNA measurements, more recent analyses (29, 30) have, in fact, found transcriptional activity to exert a dominant regulatory influence on changes in protein levels, including during active vertebrate immune responses. Moreover, we have shown that the seasonal gene expression profiles studied here correspond to experimentally determined seasonal variation in infection resistance (31).

We compared seasonal responses in the expression of immunity genes in two contrasting wild habitats and in semi-natural outdoors mesocosm habitats stocked from (and thus matched to) one of the wild habitats, replicating across 2 years. To quantify the importance of thermal effects, we continuously monitored environmental temperature within each habitat and simultaneously conducted an *in situ* manipulation of temperature in some of the mesocosms. Importantly, this allowed predictions based on the experimentally determined thermal effects to be compared with observed seasonal patterns of gene expression. To further dissect thermal effects from photoperiodic effects we also manipulated the seasonal progression of PP in a long-term laboratory experiment under different temperature conditions. The extended nature of this experiment, moreover, allowed us to assess the possibility of endogenous (clock) control. By integrating extensive field observation with experimental manipulation, we were thus able to generate compelling evidence to assess hypotheses that temperature, PP, or an endogenous circannual clock drives a seasonal fluctuation seen in the wild.

## Materials and Methods

### Overview of Study Design

We monitored environmental temperature and immune gene expression for two wild populations over 2 years. We also stocked mesocosm habitats from one of the wild localities and monitored these synchronously with the wild populations. This allowed us to describe patterns of gene expression in the wild and to establish to what extent these patterns were maintained in mesocosms. The mesocosms and wild habitats experienced equivalent PP and broadly similar temperature conditions but were subject to other habitat-specific conditions (e.g., regular provision of defined food in the mesocosms). The overall effect of these habitat-specific conditions could thus be distinguished from photoperiodic and thermal effects. Furthermore, we carried out a directional manipulation of temperature in the mesocosms. The aim of this was to estimate thermal effects on gene expression, so that we could statistically predict thermally driven expression variation in the wild (using our environmental temperature records). This allowed us to ask, quantitatively, to what degree temperature is able to explain variation seen in the wild. In addition, we carried out a laboratory experiment with a 2 × 2 factorial manipulation of temperature and photoperiodic regimen (either a natural or an accelerated seasonal PP progression). This allowed us to partition the effects of temperature and PP and also, in the absence of any photoperiodic effects, to consider the possibility of an endogenous trend. The latter could be due to an endogenous circannual clock, or to intersection with an endogenous circadian clock slightly out of synchrony with the sampling time points.

### Monitoring of Wild Populations

We monitored sticklebacks in an upland lake (FRN, 52.3599, –3.8776) and river (RHD, 52.4052, –4.0372) in mid-Wales (22). Ten fish per month were sampled from each population (±2 h of 12:00 h UTC, at regular monthly time points) from autumn to autumn in two successive years (October 2013–September 2014, December 2014–November 2015). The samples were representative of the natural cohort structure (a 0+/1+ assemblage that largely turns over to 0+ by early autumn). Within-habitat water temperatures were logged every 5 min by Tinytag Aquatic 2 (TG-4100) data loggers (reading resolution ≤0.01°C).

### Mesocosm Experiment

We stocked semi-natural outdoors mesocosms with fish from FRN and sampled these in a schedule synchronous to that for the wild populations (see above). The details of the mesocosm study have been reported in detail previously (30). Briefly, for each year’s run of the mesocosms (October 2013–September 2014, December 2014–November 2015), we stocked a different young-of-the year (0+) cohort collected at the end of the breeding season. Before the experiment, fish were exposed to two consecutive anthelmintic praziquantel treatments (24 h at 4 mg L^−1^; FlukeSolve, Fish Treatment Limited), separated by 4 days, following manufacturer’s recommendations. This removed *Gyrodactylus* spp. that might initiate epizootics detrimental to fish health (28). Fish were then acclimatized in the mesocosm system for 4–6 weeks. Mesocosms were filled with conditioned tap water and routinely run at 1% salinity as a prophylactic measure to suppress epizootics with harmful environmental pathogens such as *Ichthyophthirius*. Fish were maintained at very low biomass densities of 0.01–0.05 g L^−1^, so that absolute variation in biomass density was negligible. At the same time population sizes within each tank were sufficient for fish to undergo elective social interactions (31), e.g., shoaling. Mesocosms were arranged in a 3 × 4 array of 12 recirculating 300 L tanks covered with loosely fitting translucent lids and exposed to the open air. A 2 × 2 factorial combination of temperature and ration treatments was applied across the mesocosm tanks. For the temperature treatment, half of the tanks were left unheated, and the remainder subject to a +2°C manipulation. Heating was achieved *via* 300 W shielded heaters controlled by differential thermostats (31). The effects of this thermal manipulation on the expression of individual genes have previously been reported (31). The food treatment involved two ration levels of the same food (chironomid larvae weekly supplemented with cladocerans). This produced growth trajectories (for population mean size) that were similar to each other, and also similar to the growth trajectory in the wild at FRN (28), with a small body weight response of ~+80 mg in the higher compared with lower ration group. This treatment was not a focus of this study but is adjusted for by a factor term included in the analyses below. For the 2013–2014 mesocosm run, tanks were configured in two closed recirculating systems (heated and unheated) joining six tanks and a biological filter, in series, in each case (recirculation at 3,310 L h^−1^). For the 2014–2015 run, every tank was isolated and contained an individual stand-alone water pump unit (recirculation at 1,500 L h^−1^) with an internal 9w ultraviolet C lamp and a biological filter. In 2014–2015, continuous aeration was provided by subsurface airline feeds to each tank (~125 L h^−1^ tank^−1^). Natural plankton communities formed during the experiment that were limited, rather than ablated, by the ultraviolet treatment included in 2014–2015. Temperature in each mesocosm tank was logged every 5–10 min, to a reading resolution ≤0.05°C, by Tinytag radio temperature loggers (TGRF-3024) networked through a Tinytag Radio system. As previously described, trials using calibrated data loggers in the mesocosm systems demonstrated that the flow patterns were sufficient to disperse temperature gradients at the tank surfaces and around heaters, meaning that fish had very limited potential for temperature selection. Nitrite and nitrate levels (Tropic Marin Nitrite-Nitrate test) were continuously monitored throughout the experiment and remedial water changes carried out when nitrite levels rose above 0.02 mg L^−1^. Twenty fish per month were sampled from the mesocosm system, synchronously with sampling in the wild (see above). Each monthly sample was made up of one to two fish from each tank, taken in a pattern that equalized the numbers sampled from each tank each quarter.

### Laboratory Experiment

**Figure 1:**
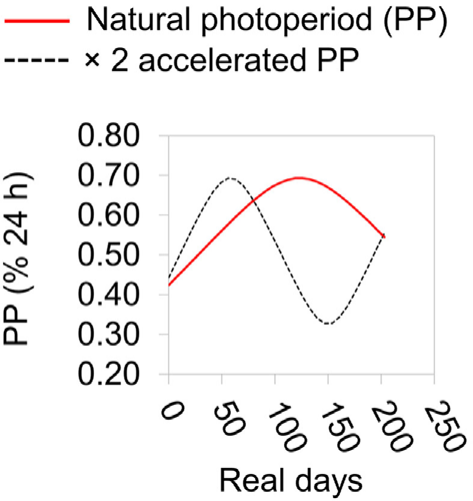
Summary of photoperiodic regimen during laboratory experiment. Photoperiod (PP) is expressed as a % of the 24 h cycle. The natural PP regimen is based on that at Cardiff, UK.

Sticklebacks were collected by hand net at Roath Brook, Cardiff, UK (51.499858°, −3.168780°) on January 6th 2015 and transported to Cardiff University aquarium. Here they were kept in 75 L tanks at a density of <1 fish L^−1^ under outdoors ambient temperature and lighting conditions. Fish were treated to remove pathogens capable of compromising fish health during the experiment (31). Initially, they were exposed to 0.004% formaldehyde solution for two 30 min periods, separated by a 30 min rest period in freshwater. They were then maintained in water at 0.5% salinity and screened for ectoparasites at least three times by briefly anesthetizing them in 0.02% MS222 and visually checking for ectoparasites under a dissecting microscope. Any ectoparasites found were removed using watchmaker’s forceps following the procedure of Schelkle et al. (32). At the beginning of the experiment (February 11th 2015), fish were assigned to factorial combinations of temperature treatment (7 or 15°C, in different CT rooms) and PP regimen treatment (natural or 2× accelerated PP regimen). During the experiment fish were kept in 8 × 30 L tanks containing water at 0.5% salinity, each with 25 fish (two tank replicates per treatment combination). Lighting was provided by fluorescent full spectrum bulbs (6,500 K) and controlled by an electric timer (±2.5 min). We assumed that sticklebacks would respond to a simple (square wave) photoperiodic cue because they have often been reported to do this in the case of reproductive cycles (23–27). Light levels were >10,000 lx during daylight periods or <10 lx during dark periods. The PP treatments were a natural seasonal day-length regimen and a regimen in which day-length change occurred in the natural sequence but was accelerated to twice the rate (i.e., a full annual day-length cycle being completed in 6 months) (Figure 1). Lighting schedule was advanced daily according to the normal daily sunrise and sunset times at Cardiff, UK (advancing 1 day/day in the natural treatment, and 2 days/day in the accelerated treatment). We chose this gradually changing regimen, as opposed to a sudden exposure to very different regimens, reasoning that the latter might induce stress effects, or disruption of circadian rhythms, that would be confounded with PP. Every week, on the same day at 12:00–13:00 h, UTC, one fish was sampled (randomly) from one of the replicate tanks within treatment combinations (alternating tanks every week) and killed and preserved as described above for wild and mesocosm fish. The experiment was continued for 30 weeks, with a final sampling point on September ninth 2015. Sticklebacks were fed daily on chironomid larvae (until satiety) at 12:00–13:00 h, following any sampling. Maintenance was in conditioned tap-water throughout.

### Sampling of Fish

For all sampling, fish were individually hand netted and immediately killed by concussion and decerebration to prevent artifacts associated with trapping or handling. Killed fish were immediately placed in RNA stabilization solution (28) and transferred to 4°C and then to −80°C for long-term storage.

### Gene Expression Measurement

Based on the transcriptomic study of Brown et al. (22) we selected 10 stickleback genes (*tbk1, orai1, il1r*-like, *gpx4a, cd8a, ighm, igzh, tirap, foxp3b*, and *il4*) at seasonally differentially expressed loci and two genes (*il17* and *il12ba*) with less definite seasonal expression. All were well expressed in both whole-fish and gill RNA pools. The roles of the products of these genes in immunity are summarized in Table S1 in Supplementary Material. We measured their expression by quantitative real-time PCR. For wild and mesocosm samples we analyzed whole-fish RNA pools, following methods previously described (22, 28), using the validated endogenous control genes *yipf4* and *acvr1l*. Samples were processed and assayed separately for each iteration of the study (2013–2014 and 2014–2015). Within each iteration samples from sampling units (site × month) were dispersed evenly across assay plates and a calibrator sample created through pooling small aliquots from all samples. Gene expression measurements from FRN, RHD, and FRN-M were thus measured on the same scale within years, allowing direct comparison. Data for wild fish in 2013–2014 and for mesocosm fish in 2013–2014 and 2014–2015 include some of those used by Brown et al. (22) or by Stewart et al. (31) in analyses with distinct objectives. All data for FRN and RHD in 2015–2016 are presented for the first time. For the PP experiment we extracted RNA from the gill (left-hand arches) employing manual homogenization and RNA Aqueous micro total RNA isolation kits (ThermoFisher), following manufacturer’s instructions. Gill tissue was used in this experiment as we have recently shown it to be especially sensitive to seasonal change and to also show similar seasonal responses to whole-fish samples (22). Different sampling units (treatment groups × time) were dispersed across assay plates, allowing statistical assessment of a plate effect, and a calibrator sample (run on all plates) created through pooling small aliquots from all samples. Other conditions were as for the whole-fish samples (above). Relative gene expression (RE) values used in analyses below are normalized to the endogenous control genes and indexed to the calibrator sample using the ΔΔCT method implemented in the real-time PCR machine (QuantStudio 12 K flex real-time PCR system; ThermoFisher) operating software.

### Data Analysis

All statistical procedures were carried out in *R* version 3.3.1 (33). We considered seasonal variation in individual gene expression variables from wild fish, initially assuming sinusoid-like variation and using cosinor regression (34–36) to provide estimates of timing (acrophase)

**Figure 2:**
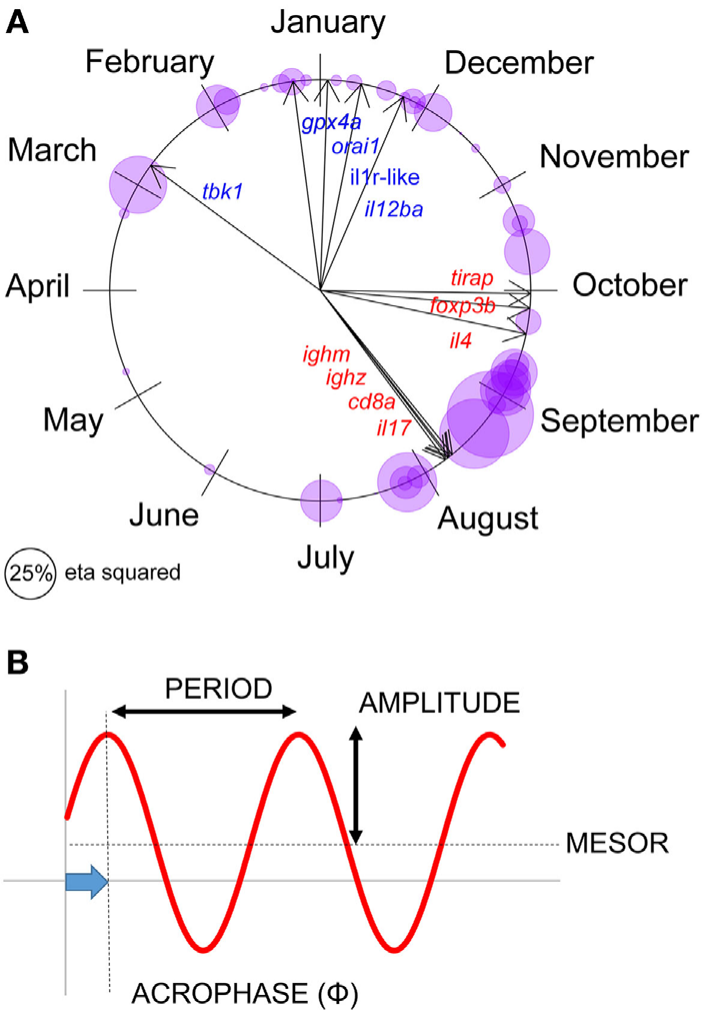
Seasonal expression responses for individual immune-associated genes in wild sticklebacks. **(A)** Circular plot of the acrophase of maximum expression in individual genes, for each site × year combination; bubbles represent individual observations and are sized according to the seasonal (sinusoid) effect size in cosinor models (classical η^2^). Arrows represent the acrophase mean direction for each gene across the two sites and years. **(B)** Parameters describing a seasonal sinusoid.

Y(t)=M+A cos (2πt/τ+ϕ)+e(t),

Y(t)=M+βX+γZ+e(t),

where *t* = time, *M* = mid-value (mesor), *A* = amplitude, τ = period (12 months), ϕ = acrophase (see Figure 2), β = *A* cos ϕ, *X* = cos(2π*t*/τ), γ = −*A* sin ϕ, *Z* = sin(2π*t*/τ), and *e* = error. The *cosinor* package was used to fit cosinor models and estimate acrophase; the same models were fitted with the *lm* command and classical η^2^ effect sizes obtained using the *heplots* package. For these analyses, the individual gene variables were optimally transformed using a Box–Cox procedure (*MASS* package). Additional to the sinusoid terms (above), we included fixed effects for sex and length (mm).

To simplify interpretation, we then constructed an additive gene expression index [seasonal reporter index (SRI)], based on prior information (22). For this, each relative gene expression variable (above) was first log_10_ transformed and standardized. The values for each gene variable were then summed, assigning negative or positive values to genes according to whether they were most expressed in winter (negative) or in summer (positive) in the transcriptomic study of Brown et al. (22).

Acknowledging the possibility that overall seasonal variation might occur in a pattern not best described by a sinusoid, we first analyzed SRI at our field and mesocosm sites in generalized additive mixed models (GAMMs) (37)

$$Y_{i}=X_{i}\beta +f(t)+Z_{i}\text{ b}+\varepsilon_{i},$$

where *Y_i_* is the response, *X_i_* is a row of a fixed effects model matrix, β is a vector of fixed parameters, *f* is a smoother function of time (*t*), *Z_i_* is a row of a random effects model matrix, b is a vector of random effects coefficients, and ε is a residual error vector.

The non-parametric smoother term in the GAMMs was used to flexibly represent temporal trends, without presupposing a particular relationship (37). All models contained a thin plate spline smoother for time, fixed effects of length and sex (male/female), and a random intercept for assay plate. In the case of the mesocosms, fixed effects for the thermal and food treatments (see above) were also included. GAMMs (with normal errors) were implemented using the *gam* command in the *mgcv* package, representing the random component as penalized regression terms. When inspection of the GAMM smoother suggested a sinusoid-like seasonal trend, we also carried out a cosinor regression, estimating amplitude and acrophase (see above). Additional to the sinusoid terms, we included fixed effects for sex and length, and also for thermal and food treatments in the case of the mesocosms.

We used the same analytical strategy (GAMM followed by cosinor regression analysis in the case of a significant temporal smoother) to secondarily consider individual gene expression metrics from the matched wild and mesocosm samples. For these analyses, the individual gene variables were optimally transformed using a Box–Cox procedure.

For analysis of gene expression variables in the PP experiment we initially compared three models (implemented with the *lm* command) to test hypotheses about the influence of PP and time. A null model contained terms for sex, length, and temperature treatment (two levels). A further model (model 1) contained the same terms as above and additionally sinusoid (cosinor) terms, cos(2π*t*/τ) + sin(2π*t*/τ), to represent a PP-independent endogenous circannual trend. A further model (model 2) additionally contained a term for PP treatment group (two levels) and its interaction with the sinusoid terms. This model represented the possibility of PP treatment effects, which might include changes of amplitude, period or phase, or loss of periodicity between groups. Individual gene variables were optimally transformed using a Box–Cox procedure for these analyses. Additional to these analyses we also searched for complex photoperiodic influences using thin plate spline smoothers in GAMMs to represent temporal trends without the *a priori* assumption of any particular functional relationship (including not assuming a fixed period). These models contained the same terms as the null model above and additionally a separate smoother for time within each level of a PP treatment factor. The difference between the group-specific smoothers was computed following the method of Rose et al. (38) to test for photoperiodic effects. Where there was no difference in the smoother between PP groups, we finally examined a GAMM model with a single smoother term to further assess the form of PP-independent temporal variation.

In formulating all of the statistical models above, we included fixed terms for sex and length throughout, as these are frequently significant in analyses of stickleback gene expression. Where we employed mixed models we initially assessed separate random terms for maintenance tank, RNA extraction batch and real-time PCR assay plate. We found that assay plate quite frequently accounted for a significant amount of variation, but that maintenance tank and RNA extraction batch did so much less frequently. As all of these three sources of variation would be expected, if important, to impact consistently on many genes (rather than inconsistently on just a few), we excluded tank and extraction batch from analyses to prevent the propagation of type I errors into analyses. To provide familiar (η^2^) effect size metrics, we present all linear (including cosinor) models without a random term for real-time PCR assay plate. However, we also inspected mixed models (fitted using the *lme4* package) including this term. In each case these provided similar inferences (and results were also corroborated in cases where we carried out GAMMs with random terms for plate, see above).

### Terminology

Seasons are defined below according to the astronomical calendar. Parameters summarizing seasonal sinusoid variation (period, amplitude, acrophase, and mesor) are defined in Figure 2.

## Results

### Consistent Seasonal Expression of Immune-Associated Genes in the Natural Environment

We first set out to confirm seasonal patterns of gene expression at our natural sites—FRN and RHD (Figure 2A). (For reference, parameters describing seasonal sinusoids are defined in Figure 2B.) We fitted cosinor regressions for each gene at each locality (Figure 2A) and inspected the estimated acrophases (reflecting timing of peak expression, see Figure 2B) and associated seasonal effect size. In many cases the seasonal effect size was large. Furthermore, the temporal distribution of peaks was bimodal, so that the mean timings for individual genes (Figure 2A) approximated to a winter–summer pattern (22). Thus, out-of-phase sets of genes were observable, with expression maxima either in the summer and early autumn, or the late autumn and winter (Figure 2A). There was no support for any expression peaks throughout the spring, or in the middle part of autumn (Figure 2A).

**Figure 3:**
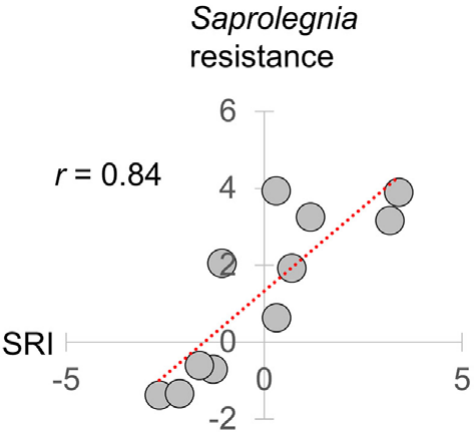
Relationship of seasonal reporter index (SRI) to an experimentally determined infectious disease phenotype. Resistance to *Saprolegnia parasitica* challenge adjusted for laboratory-determined thermal effects [the logit scale seasonal anti-*Saprolegnia* immunocompetence variable derived in Ref. (31)], plotted against mean monthly SRI. Results are based on the same 2014–2015 mesocosm run as in this study. SRI and *Saprolegnia* resistance were measured in separate groups of fish sampled contemporaneously (31). Pearson correlation coefficient (*r*) is shown in top left.

To simplify subsequent analyses, we then created an overall reporter of seasonality by calculating an additive gene expression index (SRI) of genes previously observed (22) to have winter–summer expression bias. In this index, we assigned negative values to winter-biased genes and positive values to summer-biased genes identified by Brown et al. (22) in transcriptomic data from FRN and RHD in 2012–2013 (i.e., independently from the current datasets from 2013 to 2015). Importantly with regard to its biological relevance, SRI correlated very strongly (monthly *r* = 0.84) with a previously reported (31) temperature-adjusted seasonal disease progression phenotype for the oomycete pathogen *Saprolegnia parasitica* in fish from our mesocosms (see Figure 3).

We initially analyzed SRI in confounder-adjusted GAMMs, representing temporal variation with a non-parametric smoother that made no assumption about the shape of any trend. Where sinusoid-like variation with an approximately 12-month period was observed, we then fitted a cosinor regression model to calculate the amplitude and acrophase (see Figure 2B; Table 1).

**Table 1:** Sinusoid-like circannual variation in the seasonal reporter index (SRI) of gene expression in wild (FRN and RHD) and mesocosm (FRN-M) sticklebacks.

Site/year	*N*	GAMM		Cosinor			
		Δ Dev (%)	~*P*	Ф	*A*	*P*	η^2^ (%)
FRN 2013–2014	117	32.0	1.4 × 10^−14^	−0.61 ± 0.11	3.95 ± 0.44	3.3 × 10^−14^	40.9
FRN 2014–2015	118	34.1	2.0 × 10^−16^	−0.56 ± 0.09	3.68 ± 0.33	2.2 × 10^−16^	49.7
RHD 2013–2014	112	7.3	4.0 × 10^−3^	−0.17 ± 0.23	1.81 ± 0.43	2.5 × 10^−4^	13.3
RHD 2014–2015	107	23.4	7.8 × 10^−11^	−0.07 ± 0.15	2.78 ± 0.36	4.4 × 10^−11^	36.1
FRN-M 2013–2014	230	14.1	2.3 × 10^−7^	0.72 ± 0.16	1.94 ± 0.29	1.5 × 10^−9^	15.6
FRN-M 2014–2015	216	11.6	5.4 × 10^−7^	−0.50 ± 0.13	2.39 ± 0.38	5.4 × 10^−8^	17.2

*Results for non-parametric smoother time effects in generalized additive mixed models (GAMMs) and for sinusoid time effects in cosinor regression models. For GAMMs, Δ Dev is the reduction in deviance explained by the model, in percentage points, when the time effect is deleted. The approximate P values are based on Wald tests. For cosinor regressions the estimated acrophase (Ф) and amplitude (A parameters are given ± SE, quantifying the timing and magnitude of the sinusoid. The acrophase parameter is the distance (in radians) to the summer peak from the baseline (in September). Also given is the summed classical η^2^ effect size for the cosinor (time) terms in the model*.

**Figure 4:**
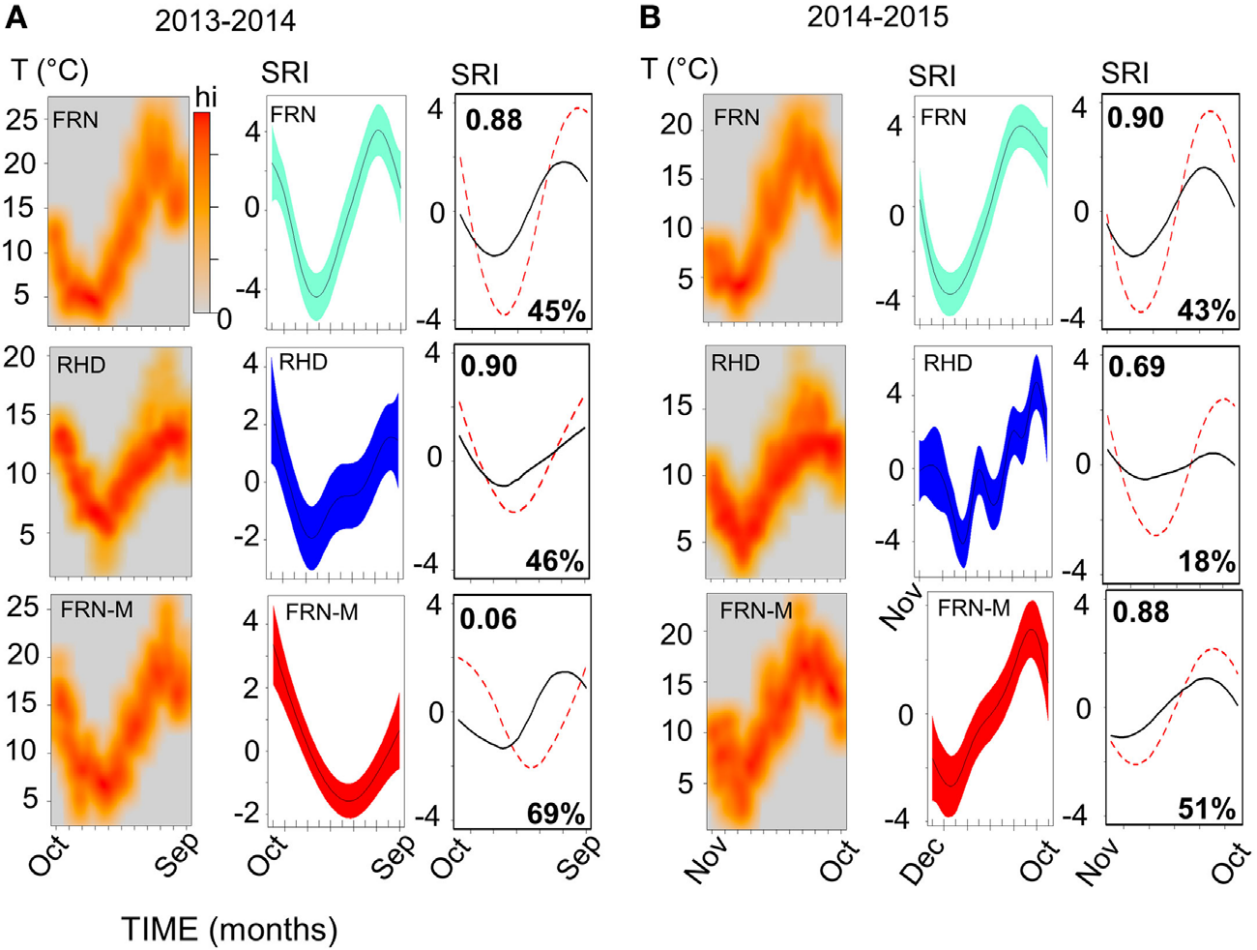
Sinusoid-like seasonal variation in the immune system of wild and mesocosm sticklebacks, as reflected by a seasonal reporter index (SRI) of expression in immune-associated genes, and its correspondence to variation in environmental temperature. Plots in **(A)**, for 2013–2014, and **(B)**, for 2014–2015, represent habitat-specific environmental temperature and SRI variation for an upland lake (FRN), a side-channel in the lowland section of a river (RHD) and semi-natural artificial mesocosm habitats stocked from FRN (FRN-M). Scatter of temperature (T) against time is plotted in the left-hand columns as a smoothed color density representation obtained through a (2D) kernel density estimate; based on recordings taken every 5 or 10 min. Middle columns show plots of SRI against time; the plotted (centered) line is a smoother from a confounder-adjusted generalized additive mixed model, on the scale of the model linear predictor, with 95% confidence interval shaded. The right-hand column shows plots of predicted SRI from a confounder-adjusted cosinor regression of SRI against time (red dotted line) and of predicted SRI from a corresponding model in which temporal sinusoid effects have been replaced by a thermal effect estimated from the experimental manipulation of temperature in the mesocosms (black line). The red dotted line thus represents observed seasonality, and the black line seasonality predicted from experimentally determined thermal effects. Correlation (Pearson, *r*) between the observed and thermally predicted values is shown in the top left-hand corner of the plots; the amplitude of the thermally predicted variation, expressed as a percentage of the observed amplitude, is shown in the bottom right-hand corner (note that the observed and predicted variation may sometimes be considerably out-of-phase, as was the case for FRN-M in 2013–2014).

**Figure 5:**
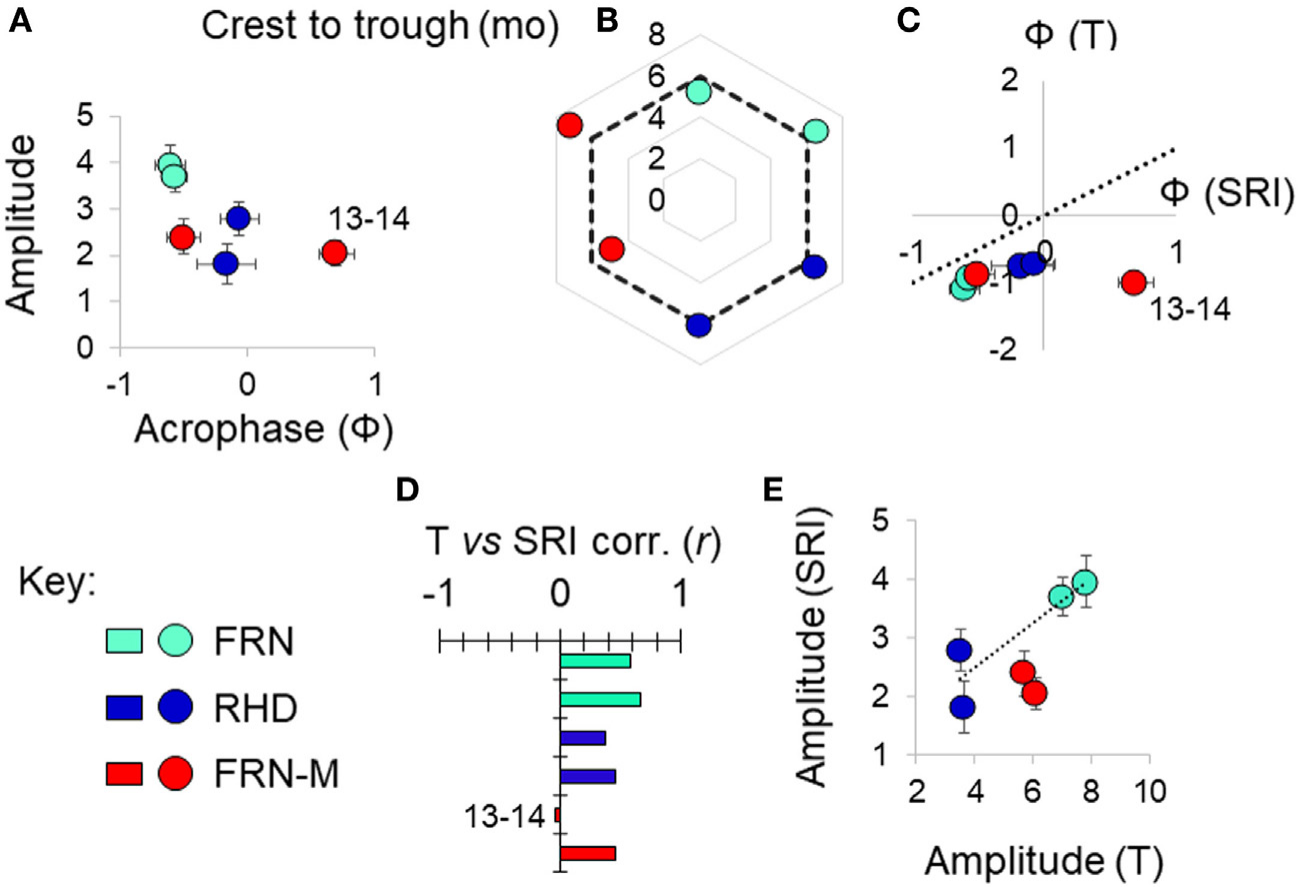
Variation in seasonal immune-associated gene expression in wild (FRN, RHD) and mesocosm (FRN-M) habitats in 2013–2014 and 2014–2015. Based on a seasonal reporter index (SRI) of expression in 12 genes. **(A)** Scatterplot of sinusoid amplitude and acrophase (Ф) of SRI variation estimated by confounder-adjusted cosinor regression; whiskers indicate 1 SE either side of estimate. **(B)** Asymmetry in the seasonal fluctuation. Radar plot shows delay between early and late season inflection points (determined graphically based on non-parametric smoother from confounder-adjusted generalized additive mixed model analysis). Dotted line indicates the symmetrical expectation given sinusoid variation; for each site, 2014–2015 points clockwise of 2013–2014 points; mo, months. **(C)** Scatterplot of acrophase for thermal variation (T) vs acrophase for SRI variation; estimates from confounder-adjusted cosinor regressions; whiskers indicate 1 SE either side of estimate. Where points are below the dotted line (T Ф = SRI Ф), there is an earlier peak for temperature than for SRI. **(D)** Pearson correlation coefficients (corr., *r*) between SRI and temperature (mean for the week prior to sampling). **(E)** Scatterplot of amplitude for SRI vs amplitude for thermal variation (°C); estimates from confounder-adjusted cosinor regressions; whiskers indicate 1 SE either side of estimate. Dotted line joins centroids for the two wild sites, for reference. **(A–E)** Sites: FRN, upland lake; RHD, lowland river side-channel; FRN-M, semi-natural mesocosms stocked with wild-caught fish from FRN. For each site, a separate datum is plotted for each study year; outlying values for FRN-M in 2013–2014 are indicated (“13–14”).

A sinusoid-like fluctuation with high SRI values in summer and low values in winter was clearly observable at both FRN and RHD in both 2013–2014 and 2014–2015 (Figure 4). These fluctuations composed a substantial component of the variation explained in statistical models (cosinor model η^2^ = 13–50%) (Table 1). The seasonal signal was much better resolved at FRN (an upland lake), explaining more variation in statistical models (η^2^ = 41–50%), than at RHD (η^2^ = 13–36%) (a minor river channel with a complex flow regimen) (Figure 4). Furthermore, there were site-specific differences in the form of the SRI sinusoid, with a larger amplitude and distinct acrophase (earlier peak) at FRN in both 2013–2014 and 2014–2015 (Figure 5A).

### Seasonal Expression of Immune-Associated Genes Is Diminished in Fish Transplanted to Semi-Natural Outdoors Mesocosms

We next compared responses in wild fish and fish transplanted to semi-natural mesocosms (FRN-M), particularly focusing on the matched comparison between FRN and FRN-M in which fish originated from the same site. Importantly, fish at FRN and FRN-M were exposed to natural PP, and so differences must result from other environmental variance. At FRN-M we found a seasonal signal resembling that at FRN and RHD (Figure 4). In comparison with the matched FRN site, this signal was much less resolved (η^2^ effect size = 16–17%, compared with 41–50%) of lower amplitude (1.5–2×) and with erratic timing (Table 1; Figures 5B,C). Notably, in 1 year (2013–2014), the seasonal oscillation at FRN-M was considerably out-of-phase with the variation seen in wild populations (FRN and RHD) (Figures 5A,C).

**Figure 6:**
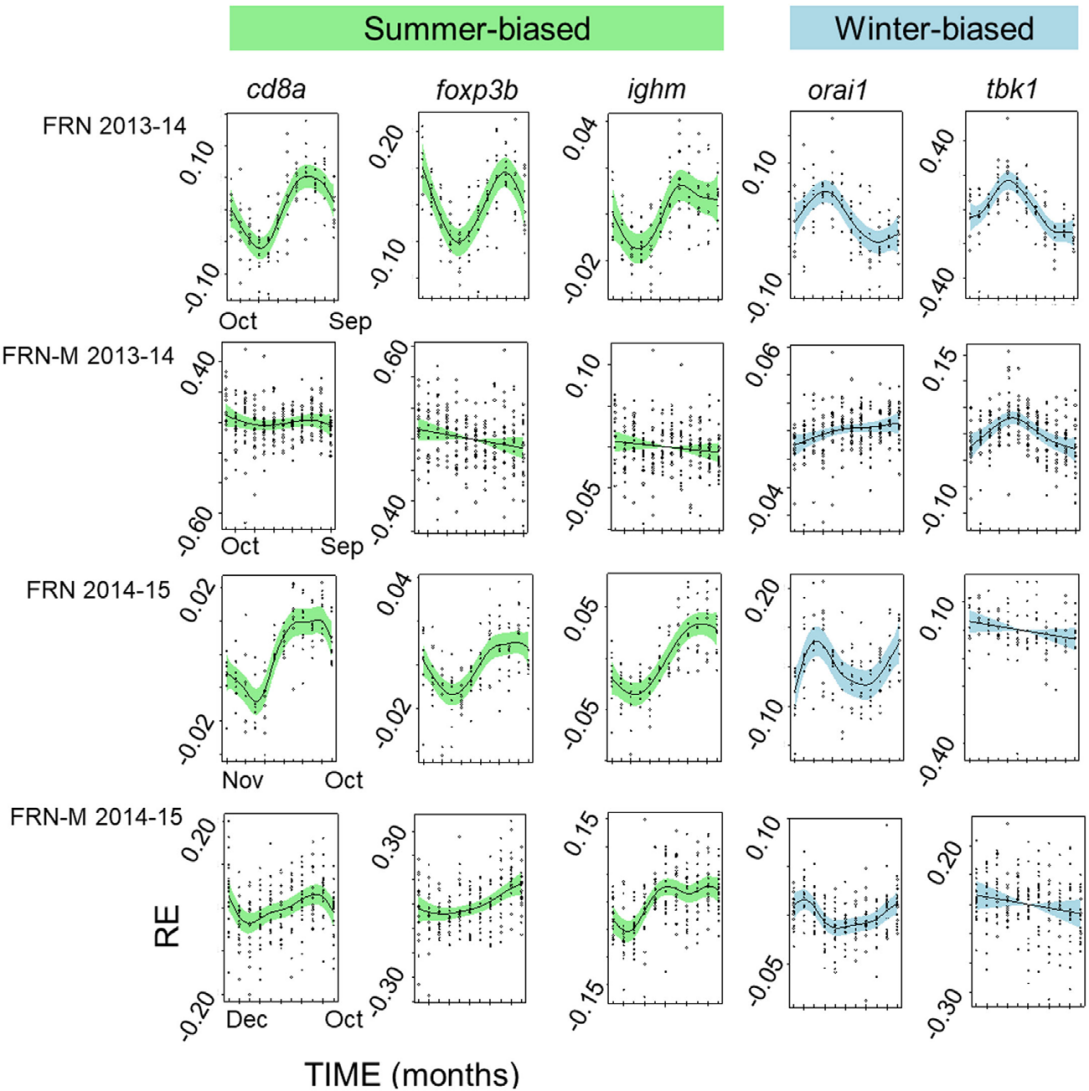
Seasonally variable expression in individual immunity-associated genes in fish from an upland lake (FRN) and from semi-natural outdoors mesocosm habitats stocked from the lake (FRN-M). Relative gene expression (RE) is shown for two annual cycles (2013–2014 and 2014–2015) based on analysis in generalized additive mixed models and plotted (centered) on the scale of the model linear predictor; lines represent non-parametric smoothers for time with 95% confidence intervals shaded and plotted points are partial residuals. Genes shown are typically relatively highly expressed in winter (winter-biased) or in summer (summer-biased) in wild habitats (22). Seasonal expression patterns are greatly diminished in the mesocosms, with inconsistent effects on different genes.

The diminution of the seasonal signal in mesocosms (FRN-M), compared with the matched wild site (FRN), was even clearer when considering seasonal expression in individual genes. To illustrate this we arbitrarily selected five genes that are consistently seasonally expressed in the wild and applied the same analytical approach as for SRI above (GAMMs followed by cosinor models, given a significant temporal smoother; see Table 2). In the wild, all of the genes showed striking sinusoid-like circannual expression trends in both years (Figure 6; Table 2), except for *tbk1* in 2014–2015. Inflection points in these trends all corresponded to the summer or winter expression biases previously reported (22). In contrast to the wild population, seasonality was much diminished in the mesocosms (Figure 6; Table 2). Only one gene (*tbk1*) in 2013–2014 and four genes (*cd8a, foxp3b, ighm*, and *orai1*) in 2014–2015 showed weak sinusoid-like annual trends, although the form of these was broadly consistent with those seen in the wild.

**Table 2:** Sinusoid-like circannual variation in the expression of individual immunity-associated genes in fish from an upland lake (FRN) and from semi-natural outdoors mesocosm habitats stocked from the lake (FRN-M).

Site/year		GAMM		Cosinor		
	Gene	Δ Dev (%)	~*P*	Ф	*P*	η^2^ (%)
FRN 2013–2014	*cd8a*	26.6	1.6 × 10^−12^	−1.14 ± 0.12 (peak)	7.3 × 10^−14^	30.0
	*foxp3b*	17.4	5.3 × 10^−7^	−0.44 ± 0.18 (peak)	1.1 × 10^−6^	19.7
	*ighm*	18.0	7.3 × 10^−10^	−1.15 ± 0.23 (peak)	2.9 × 10^−5^	16.3
	*orai1*	16.0	9.1 × 10^−4^	−1.05 ± 0.18 (trough)	1.8 × 10^−7^	21.6
	*tbk1*	38.9	2.6 × 10^−9^	−0.52 ± 0.14 (trough)	5.9 × 10^−10^	29.0

FRN 2014–2015	*cd8a*	27.8	5.8 × 10^−14^	−0.68 ± 0.12 (peak)	1.9 × 10^−14^	36.8
	*foxp3b*	10.3	1.5 × 10^−7^	−0.27 ± 0.16 (peak)	4.3 × 10^−10^	24.9
	*ighm*	14.5	1.1 × 10^−12^	−0.56 ± 0.11 (peak)	1.3 × 10^−15^	42.8
	*orai1*	10.4	0.0011	1.56 ± 0.49 (peak)	0.0438	4.7
	*tbk1*		ns			

FRN-M 2013–2014	*cd8a*		ns			
	*foxp3b*		ns			
	*ighm*		ns			
	*orai1*		ns			
	*tbk1*	8.3	3.1 × 10^−6^	−0.42 ± 0.18 (trough)	4.3 × 10^−8^	13.5

FRN-M 2014–2015	*cd8a*	9.8	6.4 × 10^−4^	−0.37 ± 0.22 (peak)	4.4 × 10^−4^	7.5
	*foxp3b*	4.5	3.2 × 10^−4^	0.06 ± 0.22 (peak)	1.4 × 10^−5^	8.3
	*ighm*	4.3	1.3 × 10^−5^	−1.06 ± 0.18 (peak)	8.3 × 10^−5^	9.1
	*orai1*	7.1	1.6 × 10^−4^	1.20 ± 0.24 (peak)	7.6 × 10^−6^	8.1
	*tbk1*		ns			

*Results for non-parametric smoother time effects in generalized additive mixed models (GAMMs) and for sinusoid time effects in cosinor regression models. For GAMMs, Δ Dev is the reduction in deviance explained by the model, in percentage points, when the time effect is deleted. The approximate P values are based on Wald tests. For cosinor regressions the estimated acrophase (Ф) parameter is given ± SE, quantifying the timing of the sinusoid. The acrophase parameters give the distance (in radians) to the closest peak or trough (indicated in parentheses) to the baseline. Also given is the summed classical η^2^ effect size for the cosinor (time) terms in the model*.

Taken together, these observations confirm that seasonal immune expression becomes weaker and more erratic in fish moved to semi-natural mesocosms. Crucially, this substantial change occurs despite the fact that mesocosms experience the same photoperiodic cues as in the wild.

### Thermal Effects Drive Seasonal Variation but Other Environmental Effects Are Also Important

We found that seasonal SRI variation approximately tracked seasonal thermal variation, but with notable discrepancies, especially in the mesocosm environment. In most cases the SRI peak lagged slightly behind that of temperature at the same site (Figure 5C) and monthly SRI correlated strongly with prevailing temperature (the mean for the preceding week; Figure 5D). This was with the exception of the 2013–2014 mesocosm run, in which the seasonal peak in gene expression was considerably delayed compared with the thermal peak (Figures 4 and 5C), and there was no correlation with temperature (Figure 5D). The site with the highest thermal amplitude (FRN) also had the highest SRI amplitude, but FRN-M, which also had a relatively high thermal amplitude, did not have a correspondingly high SRI amplitude (Figure 5E).

To achieve a clearer quantitative understanding of the importance of thermal effects we used responses to the (ambient +2°C) thermal manipulation in the mesocosm habitats (FRN-M) to predict annual thermal effects on SRI at FRN, RHD, and FRN-M. Specifically, we employed the cosinor models for SRI (above), predicting (around the mesor) for the sinusoid temporal terms and then for the estimated thermal effect applied to the habitat-specific continuous temperature monitoring data (0.249 ± 0.138/°C rise; based on a cosinor model for both years of mesocosm data with an additional term for year and interaction between the sinusoid terms and year). This allowed us to compare the observed temporal SRI sinusoid to the SRI pattern predicted by thermal measurements (Figure 4). Thermal SRI predictions underestimated the amplitude of, but were strongly correlated with, the observed SRI sinusoid at FRN and RHD. On the other hand, the predicted SRI was not always correlated with the observed SRI sinusoid at FRN-M (Figure 4).

Taken together, these results indicate that thermal variation drives a substantial component of gene expression but is insufficient to explain all of the observed seasonal variation. More specifically, it can be inferred that at FRN and RHD unidentified environmental effects acted on SRI in the same direction as temperature, augmenting thermal effects. At FRN-M, on the other hand, the effect of temperature was sometimes obscured by unidentified environmental variation that opposed, or that was less correlated with, temperature.

### Seasonal Expression of Immune-Associated Genes Is Not Explained by Year Cohort Dynamics

We considered the possibility that the seasonality we observed in the wild populations (FRN, RHD) was demographically linked, resulting from recruitment in the summer and autumn. In this scenario, if gene expression increases or decreases with host age or size this might create a seasonal fluctuation in unadjusted data. However, such an explanation was discounted by our analyses. First, seasonal oscillations like those seen in the field occurred in mesocosms (albeit in reduced form). Crucially, this occurred even though the mesocosms were stocked with a single year cohort and thus not subject to recruitment. Secondly, all analyses in the preceding section were adjusted for host length and we have previously shown length to be a substantial surrogate for age in sticklebacks from FRN (22). Moreover, even if there were a linear ontogenetic trend, the timings of seasonal oscillation in the wild do not correspond to the timing of recruitment. Thus, the winter inflection point for seasonal expression at wild sites occurs well outside the breeding season, in January or February, and a seasonal trend is visible well before recruitment occurs in the late spring and summer.

### Expression of Immune-Associated Genes Is Independent of PP and the Effect of Endogenous Timing Is Modest, at Most

We conducted a long-term laboratory experiment in which acclimated wild fish were maintained under a 2 × 2 factorial manipulation of temperature (constant 7 or 15°C) and PP. The photoperiodic treatments consisted of a (control) natural seasonal photoperiodic regimen and a 2× accelerated natural photoperiodic regimen. Fish were sampled from each treatment combination weekly for 30 weeks, a period long enough to observe at least one of the inflection points in any circannual sinusoid (such as those seen in the wild). The design enabled us to independently quantify photoperiodic and thermal effects. In interpreting possible photoperiodic effects, we considered that these would be supported by a detectable circannual oscillation in the control group accompanied by changed oscillation, or loss of oscillation, in the treatment group (including due to complex entrainment effects). In the case where a single circannual oscillation was detectable across treatment groups, this might tentatively be attributed to an endogenous rhythm (including the case of intersection with a circadian rhythm).

Most individual genes showed significant expression responses to temperature with substantial effect sizes (η^2^ = 5–15%), bearing in mind that the treatment temperatures (7 and 15°C) span less than one-third of the typical annual thermal range in the wild (Table 3). SRI also responded to temperature with a large effect size and in a direction (positive association) consistent with its seasonal variation in the field. These results, and the results of other recently reported laboratory experiments (31), are thus consistent with temperature being an important driver of immune expression in wild sticklebacks.

**Table 3:** Thermal effects (7 vs 15°C) on the expression of individual genes in the laboratory experiment (*n* = 120).

Gene	GAMM			Cosinor		
	Parameter	*P*	Δ Dev (%)	Parameter	*P*	η^2^ (%)
*cd8a*	0.0013 ± 0.0004	4.3 × 10^−4^	7.7	0.0019 ± 0.0006	1.1 × 10^−3^	8.9
*ighm*	0.0170 ± 0.0045	2.5 × 10^−4^	13.4	0.0153 ± 0.0042	4.3 × 10^−4^	10.2
*gpx4a*	0.1146 ± 0.0232	3.0 × 10^−6^	16.4	0.1117 ± 0.0244	1.2 × 10^−5^	15.3
*tirap*	0.0157 ± 0.0043	3.9 × 10^−4^	9.1	0.0165 ± 0.0049	1.1 × 10^−3^	9.0
*orai1*	−0.0036 ± 0.0009	1.9 × 10^−4^	6.2	−0.0039 ± 0.0016	0.019	4.8
*tbk1*	−0.0086 ± 0.0021	1.0 × 10^−4^	6.5	−0.0090 ± 0.0028	1.8 × 10^−3^	8.2
*il1r*-like	0.0026 ± 0.0006	3.2 × 10^−5^	7.4	0.0030 ± 0.0008	2.0 × 10^−4^	11.5

*Estimates derived from fixed terms in generalized additive mixed models (GAMMs) and in cosinor regression models. For GAMMs, Δ Dev is the reduction in deviance explained by the model, in percentage points, when the thermal effect is deleted. For cosinor regressions, a classical η^2^ effect size is given for the thermal effect. Data for individual genes for which there was a non-significant thermal effect are not shown*.

There were no significant photoperiodic or temporal effects for SRI in any of the cosinor or GAMM models we considered (Table 4). This outcome suggests that neither photoperiodic regimen, nor an endogenous clock can drive the main seasonal patterns in SRI seen in mesocosms and in the wild (see above).

**Table 4:** Cosinor regression models comparing scenarios of temporal and photoperiodic effect in the laboratory experiment (*n* = 120).

Response	Model	AIC	*P*	η^2^ (%)
SRI	(Null)	605.6		
	(1)	609.4	ns	
	(2)	610.1	ns	

*orai1*	(Null)	−267.5		
	(1)	−273.6	0.008	6.7
	(2)	−273.1	ns	

*cd8a*	(Null)	−534.2		
	(1)	−538.1	0.024	5.3
	(2)	−536.0	ns	

*ighz*	(Null)	215.4		
	(1)	211.0	0.018	5.6
	(2)	213.8	ns	

*il1r*-like	(Null)	−458.2		
	(1)	−460.7	0.045	6.5
	(2)	−456.0	ns	

*tirap*	(Null)	−13.1		
	(1)	−19.1	0.009	7.6
	(2)	−14.8	ns	

*tbk1*	(Null)	−143.7		
	(1)	−155.4	5.8 × 10^−4^	8.9
	(2)	−153.1	ns	

*Models: (Null) no temporal or photoperiodic effect, (1) a photoperiod (PP)-independent circannual effect, (2) PP-dependent effects, representing change in sinusoid form, or loss of periodicity, due to PP treatment. Akaike Information Criterion (AIC) is shown for each model and P values for F-tests between each alternative model and the preceding less complex model. There was no support for temporal or PP effects on the seasonal reporter index (SRI). Some individual genes showed a significant temporal effect, but in no case there was a significant PP effect (and no significant PP effects were detected in corresponding generalized additive mixed models). Only data for individual genes with significant effects (vs the null model) are shown above. A classical η^2^ effect size is given for the temporal effect in model (1), where this was significant*.

**Figure 7:**
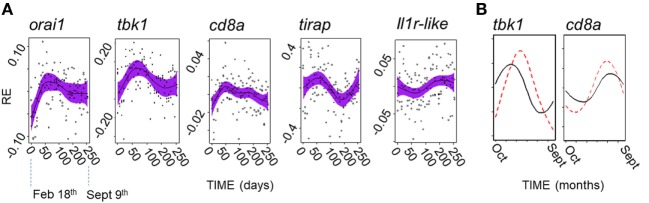
A possible endogenous oscillation in gene expression in laboratory-maintained fish (*n* = 120). **(A)** Non-parametric smoothers from generalized additive mixed models (GAMMs) (except where otherwise stated) representing temporal variation in relative gene expression (RE) for laboratory experiment running between February 11th and September 9th 2015. Timings on the *x*-axis are zeroed at the first sampling point (February 18th). Four genes show peaks (*orai1, tbk1, tirap*, and *cd8a*), and one gene a trough (*ilr1*-like) in spring (April). (Note: the smoother for *il1r*-like becomes non-significant when a random model term is added, and so represents a tentative trend only; shown is the marginally significant smoother from a generalized additive model, lacking a random term.) Solid lines show (centered) effects on scale of model linear predictor; dashed lines indicate 95% confidence interval; and points are partial residuals. **(B)** Predictions of RE given host and temperature time series data at FRN 2013–2014; based on cosinor models fitted to the laboratory experiment data, and shown for representative genes (note: *tbk1* had the highest sinusoid effect size compared with thermal effect size in the laboratory experiment). Predictions based on thermal term alone (solid line) suggest peaks with timing similar to that observed in the wild (in winter for *tbk1* and summer for *cd8a*); prediction based on the thermal and cosinor terms (dotted line) shifts peaks toward the spring.

Acknowledging the possibility of a fluctuation in gene expression profile that did not correspond to that seen in the field, we secondarily considered all of the genes that we measured individually. We found that there was no evidence of PP effects (in cosinor or GAMM models) for any gene. In contrast, 5/12 genes showed significant or near-significant sinusoid-like temporal (PP-independent) expression trends (Figure 7A) of modest effect size (η^2^ = 5–9%) (Table 4). A significant temporal trend in a sixth gene (*ighz*; see Table 4) was not sinusoid-like when analyzed in a GAMM and was not considered further. Consistently, all of the sinusoid-like trends had outlying values (peaks, four genes; troughs, one gene) in April (based on smoothers fitted in additive models, and sinusoid functions fitted by cosinor regression) (Figure 7A). Their timing was thus approximately 90° out-of-phase with the predominant winter–summer seasonality seen in the wild (above). Furthermore, the co-expression relationships among individual genes were different in the laboratory fluctuation: several genes that tended toward antiphase with each other in the natural seasonal fluctuation (i.e., either winter or summer-biased, Figure 2A) were in-phase in the laboratory (Figure 7A).

We finally asked whether the possible endogenous modality above is detectable in the field against the background of other variation. To do this we used the significant cosinor models developed from the experimental results above to make predictions for the field, which were then compared with observed variation. In the predictions, we found that the endogenous trend tended to shift the seasonal gene-specific expression peak toward the spring, when compared with a prediction based on thermal variation alone (Figure 7B). However, there was no evidence for spring-wards shifts in the wild fish gene expression data, which corresponded quite closely to the thermal prediction. In fact, in the wild, the spring and early summer period was devoid of well supported seasonal peaks for individual genes (Figure 2A). Instead, and contrary to expectation based on the laboratory endogenous trend, where peaks did not occur in winter or summer they occurred in early or late autumn (Figure 2A). Moreover, SRI variation tended to be close to the thermal prediction in the wild, but always displaced toward autumn rather than the spring (Figures 4 and 5C). Hence, these results suggest that the effect of temperature, in combination with other unknown environmental drivers, overwhelms any endogenous circannual variation in natural conditions.

## Discussion

Using a combination of field, mesocosm, and laboratory experimental observations, we have demonstrated that photoperiodic control of seasonal immune allocation in sticklebacks is negligible (despite the well-established photoperiodic control of reproduction). Moreover, any variation due to endogenous rhythmicity is modest, at most, and out-of-phase with the predominant pattern of seasonality seen in the field. We have, furthermore, shown that thermal effects on immune allocation are substantial and can drive circannual oscillations approximately in-phase with those seen in nature (overwhelming any endogenous rhythmicity). Importantly, however, these thermal effects appear to be readily overridden themselves by other, unidentified, environmental variation.

Such results are of wider interest because seasonal patterns of immunity have been reported in many vertebrate systems (7, 12), and yet their control is incompletely understood. Importantly, such seasonal responses likely influence the dynamics of infectious disease (2–4), and contribute to individual health and fitness. Understanding their origin may help to link individual heterogeneity in within-host disease progression and between-host disease transmission to predictive environmental measurements, increasing the possibility of projecting disease risk. In relation to climate variation, furthermore, the nature of the cues that control seasonal phenotypes are likely to affect resilience to rapid climate change in naturally occurring organisms. Thus, where a species has evolved fixed responses to unvarying predictors of season (e.g., molecular clocks or astronomical signals such as PP), as is sometimes the case (5, 10), this could reduce resilience as adaptation may have to occur through molecular evolution rather than plasticity. On the other hand, where organisms respond plastically to seasonal variables that directly constrain their exploitation of the environment (39), as we have mainly found here, they may be better preadapted and resilient to change.

Based on a genome-wide transcriptomic analysis we have previously observed (22) a marked circannual oscillation of immune-associated gene expression in wild *G. aculeatus*. This oscillation is represented by two distinct sets of genes with differing expression periodicity: with expression in one (summer-biased) set being out-of-phase with that of another (winter-biased) set. In the summer-biased set are many genes involved in adaptive effector responses, while the winter-biased set lacks such genes but contains many innate genes and genes linked to regulation or suppression of lymphocyte proliferation (22). Moreover, we have previously demonstrated (31) a link between this seasonal gene expression pattern and winter-biased infectious disease progression. In this study, we utilized 12 genes [identified in the transcriptomic study of Brown et al. (22)] as reporters of seasonality, combining them into an expression index (seasonal reporter index, SRI) that was maximized at the expected summer expression pattern (i.e., assigning negative values to winter-biased genes and positive values to summer-biased genes). Using this index, we confirmed clear winter–summer sinusoid-like seasonality in two different annual cycles (2013–2014, 2014–2015) in all of the habitats in our field experiment: two wild localities and semi-natural mesocosms.

There was considerable variation in the signal strength, amplitude and timing of SRI sinusoids in different habitats, and between years in the case of the mesocosm populations. In the wild lake habitat the seasonal signal was more resolved, and of higher amplitude, than in the wild river locality and the semi-natural mesocosms. As all of the habitats experienced the same photoperiodic regimen, and the lake and mesocosm fish were of the same genetic origin, this variation between sites and years must be driven by habitat- and year-specific seasonal effects, perhaps including thermal effects (31). In fact, the magnitude of crude correlation between the SRI and prevailing temperature varied between strong (mostly) and very weak. Importantly, we were able to gain additional insight through the response to our manipulation of temperature in the mesocosms, and the fact that gene expression was measured in wild and mesocosm fish on the same scale as part of a regular sampling design. This allowed us to statistically predict SRI variation from our field monitoring of temperature at all sites and to quantitatively compare these predictions with observed patterns. The comparisons suggested that, in all habitats, temperature variation predicted a smaller fluctuation than observed. Furthermore, the predicted fluctuation was generally synchronous with the observed fluctuation, but could be considerably out of synchrony in the mesocosms. Hence, in the mesocosms, non-thermal seasonal environmental influences must at times counteract thermal effects, resulting in the observed asynchrony. On the other hand, in the lake and river, and at other times in the mesocosms, the effects of temperature may be augmented by other non-thermal (31) seasonal environmental influences acting in unison (in-phase) and resulting in observed fluctuation that is synchronous with, but greater than, thermal predictions. Thus, we demonstrated that temperature can drive substantial seasonal fluctuations like those seen in the field, but that a significant (and variable) component is independent of temperature and driven by other environmental variation.

Interestingly, the diminution of seasonality in the mesocosms compared with the (matched) lake habitat was even more apparent when considered at the level of individual genes. Where there was a partial loss of seasonality, this affected some genes more than others, in a site × year dependent way. For example, when we compared particularly consistently seasonally expressed genes (*tbk1, orai1, ighm, cd8a*, and *foxp3b*) between lake and mesocosm we found clear seasonality with the expected winter or summer maximum in the lake fish (9/10 gene × year instances). This was with one exception, *tbk1* in 2013–2014, for which there was, singularly and contrary to the general pattern, no seasonality. In contrast to the lake habitat, seasonal patterns were detectable in much fewer (5/10) instances in the mesocosms. This was only for *tbk1* in 2013–2014, and for *cd8a, foxp3b, ighm*, and *orai1* in 2014–2015. Moreover, although still broadly approximating the expected winter–summer oscillation, these seasonal patterns were indistinct compared with those seen in the lake. Taken together, the complexity of the gene-specific patterns observed, where some genes may maintain seasonal expression while others do not, is indicative of a multifaceted cross talk between the environment and immune system. This is consistent with a multifactorial environmental control involving not just temperature, but also other environmental drivers (as developed above) that might act through different regulatory mechanisms and pathways.

Our laboratory experiment allowed us to partition the effects of PP and temperature under otherwise constant conditions. The results confirmed a lack of response to PP, which thus cannot drive the major summer–winter fluctuation seen in the field. Given this lack of photoperiodic effect, the long-term nature of the experiment also enabled us to exclude the possibility that an endogenous circannual oscillation might contribute to the major winter–summer variation seen in our field studies. Moreover, the design allowed us to exclude that the major field variation was due to an intersection of our monthly field sampling schedule with a circadian rhythm (e.g., where the phase point for the circadian rhythm might shift relative to the monthly sampling points, giving the appearance of a long-term rhythm). Thus, while our study was designed with sampling points close to 12:00 (UTC) so that they occurred in approximately the middle of day time and minimized the chance of such an effect, any notional circadian influence could be ruled out if no substantial pattern similar to that in the wild was observed in the laboratory experiment. In fact, we only detected a very modest sinusoid-like temporal trend, with different timing and phase relationships of individual genes to the summer–winter fluctuation seen in the field. This confirmed that the major pattern seen in the field cannot be due to an endogenous circannual rhythm or to intersection of our monthly sampling with a circadian rhythm.

The small endogenous fluctuation seen in the laboratory experiment involved 5/12 genes and was approximately 90° out-of-phase with the observed major natural oscillation. In the laboratory trend, most reporter genes (regardless of their summer bias or winter bias in the field) responded in the same direction (4/5), with highest expression values in April. While this modality was smaller than the variation driven by temperature (see below), its timing suggests that it could possibly represent immunophenotypic adaptation to cope with the onset of the breeding season. For example, the predominant upregulation of immune-associated genes in April might reflect a need to reinforce immunocompetence in anticipation of increased transmission and stress during aggregation and social interactions. However, further studies are required to characterize this fluctuation, as it has only been observed once, and to confirm that it was not an undetermined experimental artifact.

We note that in our laboratory experiment we assumed that any photoperiodic control of immune allocation in sticklebacks would respond to changes in a square wave photoperiodic regimen. While it is now recognized that spectrally distinct twilight periods in the natural day–night light cycle may provide additional cues entraining circadian and circannual patterns in some vertebrates (40–42), it seems unlikely that a lack of simulated twilight would ablate photoperiodic control in the case of sticklebacks. Thus, the above assumption is reasonable because reproductive activity in sticklebacks has frequently been shown to respond to square wave photoperiods, whether a twilight is additionally simulated (26, 27) or not (23–25, 43, 44), and independent of light wavelength (45).

Significant thermal effects were recorded for a majority of genes in the laboratory experiment, including all genes involved in the endogenous trend above. This corresponded to a larger effect size (in the context of the natural temperature range) than for the endogenous oscillation. Nevertheless, predictions based on the laboratory experiment effects (applied to field datasets) suggested the endogenous oscillation, when occurring alongside thermal effects, would push annual peak expression values spring wards. In contrast, observed variation at all our sites contradicted this possible trend. There was a deficit of genes with well supported peak expression from April to June. Furthermore, where genes departed from the predominant pattern of winter- or summer expression bias, they tended to peak in early or late autumn. It was also the case that in the only year × habitat combination where SRI departed from a summer peak close to the thermally predicted peak (mesocosms in 2013–2014), this peak was, in fact, shifted toward autumn and not spring. These facts suggest that, in practice, the combination of thermal variation and other environmental drivers was sufficient to overwhelm any endogenous oscillation.

Taken together, the above pattern of results throws crucial new light on the nature of thermal control of immune allocation. The responses to temperature that we observed may anticipate reduced efficiency of certain functional responses at low temperature [for example, impaired lymphocyte function (46)]. Or they may prepare for constraints imposed by wider environmental conditions associated with lower temperature (for example, limitation of feeding or nutrient assimilation, or altered pathogen proliferation or transmission). Importantly, despite the strength of the thermal influence on immune allocation, this was sometimes overridden by other environmental variation (as in the 2013–2014 mesocosm run). This is consistent with thermal cues exerting their effects through active, context-dependent regulatory controls, rather than passively, simply through reducing kinetic energy available for molecular processes. Such an active control is independently supported by our recent finding that the immune-associated stickleback genes whose expression increases in winter include a set of genes regulating or suppressing adaptive immune responses (22).

In conclusion, our results provide compelling evidence that the direct control of circannual immune allocation *via* photoperiodic time measurement is negligible in a teleost fish, and thus not an evolutionarily conserved feature in all vertebrates. Although a small component of seasonal variability may be controlled by an endogenous oscillator, the effect size of this is, at most, very modest. Importantly, we demonstrate, also with compelling evidence, that while temperature can be a substantial driver of immune allocation in the wild, its immunomodulatory effects are readily overridden by other environmental variation. Having accounted for a large component of seasonal immune variation here, our future studies will attempt to reveal the remaining components (e.g., due to infection pressures, nutrition, abiotic conditions) using a combined observational and experimental approach. Very importantly, our present observations add to evidence that immune allocation in fish responds to thermal variation as a strategic (and overridable) cue, rather than just being constrained by it through biochemical kinetics. This points to the existence of temperature-sensitive immunoregulatory mechanisms that might be conserved in other vertebrates (47–50).

## Ethics Statement

Use of animals conformed to U.K. Home Office (HO) regulations. Elements at Aberystwyth University did not involve HO regulated procedures and were approved by the animal welfare committee of the Institute of Biological, Environmental and Rural Sciences (IBERS), Aberystwyth University and conducted following consultation with the HO inspectorate. Elements at Cardiff University were approved by the Cardiff University Animal Ethics Committee and conducted under HO License PPL 302876.

## Author Contributions

AS contributed to the design of, and carried out, the laboratory experiment and contributed to analysis of data and writing the paper. PH contributed to the design and conduct of molecular assays and fieldwork and to writing the paper. HW contributed to the design and conduct of molecular assays. MB contributed to the design and conduct of molecular assays and fieldwork. IF contributed to the design and conduct of molecular assays and carried out fieldwork. JC contributed to applying for funding, management of research, design of the laboratory experiment, and to writing the paper. JJ contributed to applying for funding, management of research, design of the laboratory experiment, design of the fieldwork, design of the molecular assays, analysis of data, and to writing the paper.

## Conflict of Interest Statement

The authors declare that the research was conducted in the absence of any commercial or financial relationships that could be construed as a potential conflict of interest.
