# Supplementary material for: Physical Cues Controlling Seasonal Immune Allocation in a Natural Piscine Model
Source: Front Immunol. 2018 Mar 22;9:582. doi: 10.3389/fimmu.2018.00582 (PMC5874293; doi:10.3389/fimmu.2018.00582)
Supplement: Supplementary file 1 [file Table_1.docx]

| **Gene** | **Role of gene product in immunity** |
| --- | --- |
| *cd8a* | Cytotoxic T cell responses |
| *foxp3b* | Regulatory T helper cell responses |
| *orai1* | T cell activation |
| *tbk1* | Induced innate antimicrobial responses |
| *il1r*-like | Inflammatory responses |
| *ighm* | Antibody responses (systemic) |
| *ighz* | Antibody responses (secretory) |
| *il12ba* | T-helper cell type 1 (Th1) responses |
| *il17* | T-helper cell type 17(Th17) responses |
| *il4* | T-helper cell type 2(Th2) responses |
| *tirap* | Induced innate antimicrobial responses |
| *gpx4a* | Anti-oxidative activity correlated to broad immune activity |

**Supplementary Table S1.** Panel of genes for which expression measurements were made by real-time quantitative PCR (Q-PCR) and the role of the products of these genes in immunity. See Hablützel et al. (2016) for further details.

Hablützel, P.I., Brown, M., Friberg, I.M., and Jackson, J.A. Changing expression of vertebrate immunity genes in an anthropogenic environment: a controlled experiment. *BMC Evol. Biol.* (2016) **16**:1-12.
